# Confocal Microscopy and Anterior Segment Optical Coherence Tomography Imaging of the Ocular Surface and Bleb Morphology in Medically and Surgically Treated Glaucoma Patients: A Review

**DOI:** 10.3390/ph14060581

**Published:** 2021-06-18

**Authors:** Carmela Carnevale, Ivano Riva, Gloria Roberti, Manuele Michelessi, Lucia Tanga, Alice C. Verticchio Vercellin, Luca Agnifili, Gianluca Manni, Alon Harris, Luciano Quaranta, Francesco Oddone

**Affiliations:** 1 IRCCS-Fondazione Bietti, Rome, Via Livenza, 3, 00198 Rome, Italy; carmela.carnevale@fondazionebietti.it (C.C.); gloria.roberti@fondazionebietti.it (G.R.); manuele.michelessi@fondazionebietti.it (M.M.); lucia.tanga@fondazionebietti.it (L.T.); gianlucamanni53@gmail.com (G.M.); 2 Department of Surgical & Clinical, Diagnostic and Pediatric Sciences, Section of Ophthalmology, University of Pavia—IRCCS Fondazione Policlinico San Matteo, 27100 Pavia, Italy; ivano.riva@virgilio.it (I.R.); luciano.quaranta@unipv.it (L.Q.); 3 Department of Ophthalmology, Icahn School of Medicine at Mount Sinai Hospital, New York, NY 10029, USA; alice.verticchio@gmail.com (A.C.V.V.); palonharris@gmail.com (A.H.); 4 Ophthalmology Clinic, Department of Medicine and Aging Science, University G. d’Annunzio of Chieti—Pescara, 66100 Chieti, Italy; l.agnifili@unich.it; 5 Department of Clinical Sciences and Translational Medicine, University of Rome Tor Vergata, Viale Oxford 81, 00133 Rome, Italy

**Keywords:** glaucoma, ocular surface, medical treatment, surgical treatment, in vivo confocal microscopy, anterior segment optical coherence tomography

## Abstract

Glaucoma patients often suffer from ocular surface disease (OSD) caused by the chronic administration of topical anti-glaucoma medications, especially in cases of long-term therapy with preserved or multiple drugs. Additionally, glaucoma surgery may determine ocular surface changes related to the formation and location of the filtering bleb, the application of anti-mitotic agents, and the post-operative wound-healing processes within the conjunctiva. Recently, several studies have evaluated the role of advanced diagnostic imaging technologies such as in vivo confocal microscopy (IVCM) and anterior segment-optical coherence tomography (AS-OCT) in detecting microscopic and macroscopic features of glaucoma therapy-related OSD. Their clinical applications are still being explored, with recent particular attention paid to analyzing the effects of new drug formulations and of minimally invasive surgical procedures on the ocular surface status. In this review, we summarize the current knowledge about the main changes of the ocular surface identified at IVCM and AS-OCT in glaucoma patients under medical therapy, or after surgical treatment.

## 1. Introduction

Glaucoma is a chronic optic neuropathy characterized by death of retinal ganglion cells and their axons, leading to corresponding visual field loss [1]. It is estimated that approximately 76 million people have glaucoma in 2020 and it is expected that the number will increase to 112 million by 2040 [2].

Glaucoma is a multifactorial disease in which an elevated intraocular pressure (IOP) has been identified as the major risk factor for disease onset and progression to blindness [3,4]. Therefore, the main purpose of treatment is to preserve the patient’s visual function and quality of life (QoL) by lowering IOP with medical, laser, and/or surgical therapies [5,6].

According to the European Glaucoma Society Guidelines, the first approach to control IOP is topical monotherapy [7]. However, up to 50–75% of patients require a combined therapy with two or more drugs in order to reach their target IOP with consequent effects on the health status of the ocular surface [5,8,9].

Long-term use of antiglaucoma drugs, history of therapy changes due to ocular surface intolerance, repeated daily instillations, and the action of active compounds and preservatives may result in the onset of the ocular surface disease (OSD), which can compromise the patient’s adherence to therapy, satisfaction, QoL, and treatment outcome [10,11,12]. Moreover, the drugs induced alterations of the ocular surface and may increase the rate of failure of glaucoma filtration surgery, which, unfortunately, may further contribute to the OSD worsening [13,14].

OSD is a multifactorial symptomatic disorder characterized by an imbalance in the homeostasis between the tear film and the ocular surface morpho-functional unit with the involvement of the conjunctiva, cornea, Meibomian glands (MGs), and lacrimal glands [15,16]. It has been reported to affect approximately 15% of the general elderly population [17] and 48% to 59% of medically treated glaucoma patients [18,19].

Reported symptoms of OSD are dryness, grittiness, burning, irritation, itching, tearing, foreign body sensation, transient visual disturbance, and blurred vision [11].

Signs are conjunctival hyperemia, modifications in tear film stability and osmolarity, eyelid inflammation, and toxic or immune-allergic blepharitis related to MGs’ dysfunction [20,21]. In addition, OSD is characterized by many subclinical changes relevant to ocular surface physiology such as a decrease in the density of goblet cells (GCs), inflammatory cells’ infiltration, and a reduction in corneal sensitivity and in the number and density of the corneal sub-basal nerve fibers [21].

In clinical settings, the diagnosis of OSD is based on slit lamp examination, tear film break-up time [22], Schirmer test score, and corneal and conjunctival staining [23].

Additionally, ex vivo histologic techniques and impression cytology are useful, even in asymptomatic patients, to identify an abnormal expression of interleukins and inflammatory markers [24].

However, ex vivo techniques are significantly invasive, and detailed morphological and quantitative analysis of the ocular surface microstructures are better performed using dedicated ophthalmological imaging platforms.

In vivo confocal microscopy (IVCM) is a diagnostic tool able to perform in vivo high-resolution ocular tissue images in real time. IVCM provides accurate microstructural information of the ocular surface and adnexa at the cellular level in a quick and non-invasive manner with a resolution comparable to that of histologic methods [15,25].

In the last years, the advent of new generation anterior segment optical coherence tomography (AS-OCT) in clinical practice allowed to obtain additional valuable information at the tissue level, including the ocular surface epithelia and the tear film, in a noninvasive way [26].

In this review, we describe the main detrimental effects induced by medical and surgical glaucoma therapy on the ocular surface, with particular attention to their appearance at IVCM and AS-OCT.

## 2. Methods

A literature review was performed using the PubMed Database, which was searched using the following phrases: ocular surface, glaucoma, ocular hypertension, medical therapy, preserved and preservative-free glaucoma medications, glaucoma filtration surgery, IVCM, and AS-OCT. In total, this research yielded more than 130 publications. All available abstracts were reviewed for relevancy to the specific topic under discussion and 115 articles were deemed appropriate to be included. Detailed information was extracted into a summary outline and then expanded to produce this review. Non-English language studies and papers irrelevant to the ocular surface and medical and surgical glaucoma management were excluded.

## 3. Medical Treatment

### 3.1. Ocular Surface and Medical Therapy: Subclinical Inflammation

Five different classes of medications are available for the topical management of glaucoma, including β-adrenergic antagonists, prostaglandin analogs (PGA), cholinergic agents, adrenergic agonists, and carbonic anhydrase inhibitors, each demonstrating varying acceptable levels of safety and efficacy [11].

The mechanisms by which antiglaucoma drugs have an effect on the ocular surface may be either allergic or toxic. The former is usually detected at the initiation of therapy, whereas the latter is more frequent and related to the chronic action of both active compounds and preservatives [27,28].

Benzalkonium chloride (BAK), a quaternary ammonium compound with bacteriostatic, bactericidal, and surfactant properties, is the most common preservative used in ophthalmic preparations including IOP-lowering medications [29].

Its effects on the ocular surface have been extensively studied in both preclinical and clinical studies [30]. BAK induces time- and concentration-dependent cytotoxic effects on the ocular surface cells characterized by inflammatory cell infiltration, overexpression of class II antigens, adhesion molecules, chemokines, chemokine receptors, interleukins, reduction in GCs’ density, expression of cell death markers, and induction of apoptosis [30,31].

In more detail, the administration of preserved latanoprost or timolol eye drops was associated with an increased expression of cytokines such as interleukin-6, interleukin-8, and interleukin-10 in the conjunctival epithelial cells [32]. Additionally, in patients receiving topical beta-blockers, corneal staining, conjunctival GCs’ loss, and squamous metaplasia have been shown [33], with the frequency of these subclinical effects reported to be higher with preserved than with preservative-free (PF) eye drops [32,34].

However, the direct effects of the active compounds cannot be ruled out. Baudouin et al. [35] reported that the expression of human leucocyte antigen-DR, a marker of inflammation, was higher in patients treated with preserved drugs or multitreatment than in untreated eyes, and it was slightly more elevated in the PF group than in the control group, suggesting a low level of subclinical inflammation induced by the active compound.

Moreover, topical application of 0.005% PF latanoprost on murine eye induced ocular surface inflammation, including activation of P38-NF-кB pathway, production of inflammatory cytokines, and CD4+ T cells’ infiltration [36], and promoted cell apoptosis; in a more macroscopic way, it decreased tear production, induced conjunctival GCs’ loss, and disrupted the corneal epithelial barrier [36].

### 3.2. Morphologic Changes: In Vivo Confocal Microscopy

**Figure 1:**
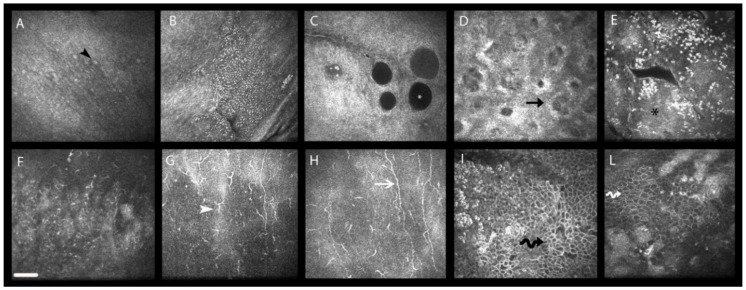
In vivo confocal microscopy (IVCM) of the ocular surface tissues in multi-treated medically controlled glaucoma. (**A**–**E**) Confocal frames taken from a patient controlled with a preserved fixed combination of timolol and dorzolamide and a preservative-free prostaglandin analog (PGA) (three eyedrops per day). (**A**) Goblet cells (GCs). GCs appear as hyper-reflective elements (black arrowhead) dispersed within the epithelium, and often appear scattered with an evident reduction of their density. (**B**) Inflammatory infiltrates. Inflammatory infiltrates appear as clusters of small hyper-reflective and mono-nucleate elements (presumably lymphocytes) infiltrating the epithelium of the tarsal or bulbar conjunctiva. (**C**) Epithelial microcysts. These structures (white asterisk) represent hallmarks of aqueous humor outflow stimulation through the uveo-scleral route, rather than detrimental effects induced by medications. (**D**) Meibomian glands. These glands appear markedly reduced in their dimension, with hyper-reflectivity of acinar wall and interstice; the black arrow indicates a glandular acinus. (**E**) CALT. Roundish immune follicles (black asterisk) appear infiltrated by numerous small hyper-reflective mono-nucleate cells (presumably lymphocytes). (**F**–**L**) Confocal frames taken from a patient controlled with a preserved fixed combination of timolol and dorzolamide, PGA, and brimonidine (five preserved eyedrops per day). (**F**,**L**) Limbal transition epithelium. The transition epithelium of the limbus appears irregular with scattered and highly hyper-reflective inflammatory elements (**L**), and with evident features of cellular polymegathism (undulated white arrow). (**G**–**I**) Cornea. The sub-epithelial layer and the Bowman’s membrane (**G**,**H**) present infiltration and activation of numerous dendritic cells (white arrowhead), with alterations of sub-basal nerve plexus morphology (white arrow). The corneal epithelium (**I**) appears markedly irregular with a higher degree of cellular polymorphism and polymegatysm. Bar represents 50 µm.

In the last two decades, IVCM has been progressively used to study the sub-clinical and clinically relevant changes of the ocular surface in patients with medically controlled glaucoma [24] (Figure 1) (Table 1 and Table 2).

Several structures can be analyzed including bulbar and palpebral conjunctiva, cornea, limbus, and MGs; in detail, it has been used to the examine the GCs’ and MGs’ density, the Vogt’s palisades and transition epithelium of the corneoscleral limbus, corneal epithelial and endothelial cell density, stromal keratocytes, and several parameters related to the sub-basal corneal nerve plexus [37].

#### 3.2.1. Conjunctiva

Antiglaucoma drugs may induce modifications of the conjunctiva from the epithelium to stroma.

Ciancaglini et al. described at IVCM the presence of epithelial microcysts in the bulbar conjunctiva in untreated ocular hypertension (OH) and in topically treated primary open-angle glaucoma (POAG) patients [38]. A higher microcyst density and area were found in patients on combined β-blocker/prostaglandin therapy when compared with PGA monotherapy, suggesting that combined therapy may enhance the aqueous humor outflow more than monotherapy [38]. Moreover, the authors did not exclude that a higher BAK concentration in the combined therapy could be a contributing factor for the increased epithelial microcysts density and area [38].

The presence of epithelial microcysts may have different explanations: they may be considered an adaptive mechanism in eyes with reduced aqueous humor trabecular outflow, but also a sign of degenerated GCs, or a hallmark of epithelial disruption [25,39].

Other epithelial changes found with IVCM are squamous metaplasia, desquamation, keratinization, thickening, subepithelial fibrosis, conjunctiva-associated lymphoid tissue (CALT) activation, dendritic cells’ (DCs) activation, and GCs’ loss [40,41].

GCs are essential for the mucin production, which guarantee the adhesion between the tear film and epithelia. Therefore, GCs’ loss decreases mucin production, and thus the tear film stability; this leads to a reduced tear clearance, with the development of dry eye and conjunctival inflammation [25].

To date, few studies were specifically conducted to determine the effects of distinct classes and regimen of topical drugs on the density of GCs in patients with glaucoma with different results. Ciancaglini et al. [42] found a marked decrease of GCs and a worse score of epithelial regularity in patients receiving preserved levobunolol with respect to those treated with the unpreserved formulation, suggesting the toxicity of preservatives on the ocular surface. Similarly, Frezzotti et al. [43] reported a significant increase in the cumulative grading score of epithelial irregularity and a reduction in the intra-epithelial GCs’ density at IVCM after 12 months of treatment with preserved timolol; conversely, these changes did not occur in controls and patients treated with PF timolol 0.1% gel formulation. However, even if the duration of both studies was limited to a short period of time (6 and 12 months, respectively) [42,43], it is possible to suppose that preservatives could exert their toxic effect on the conjunctiva after a few months of therapy.

Differently, PGAs seem to have distinct effect on GCs [44]. PF tafluprost increased GCs density until the sixth month in naïve glaucomatous patients, suggesting the aptitude of prostaglandin derivatives to stimulate mucin secretion [45]. An increase in GCs’ density at IVCM was also reported in patients receiving bimatoprost 0.01% with respect to those treated with the bimatoprost 0.03% formulation [46]. This result seems to suggest that the concentration of the active drug is more influential than the concentration of BAK in the tolerability of chronic therapy. However, a weakness of this study is represented by its open-label design and by the strict inclusion criteria that allow to analyse the safety of bimatoprost and BAK only in the glaucomatous eyes with relatively healthy ocular surface compared with the average glaucoma patient usually being treated [46].

Additionally, less pronounced conjunctival modifications were found in patients treated with preservative PGA, in comparison with patients controlled with other classes of IOP lowering medications. Specifically, PGA induced a lower reduction in GCs’ density when compared with other drug classes and no changes in the subepithelial collagen fiber diameter and expression of subepithelial fibrosis when compared with controls [47]. Moreover, GCs’ loss is even more frequent in patients treated with two drugs compared with patients in monotherapy [41].

Conjunctival stromal changes, generally less frequently observed than epithelial modifications, are represented by infiltration of inflammatory cells, fibroblasts proliferation, and activation with connective tissue deposition [40].

Finally, IVCM allows to study the effects of medications on CALT; this structure is mainly located in the lamina propria of the tarsal conjunctiva and characterized by a layer of specialized secretory lympho-epithelium, lymphoid follicles, crypt-associated lymphoid structures, and high endothelial venules [48]. At IVCM, the follicular CALT appears as defined round structures hosting hyper-reflective cellular elements in a collagen scaffold [48,49].

Confocal studies, conducted on animals, documented an intrafollicular infiltration of inflammatory cells after instillation of BAK alone or BAK-preserved PGA, whereas the PF-PGA did not activate the conjunctival immune response [50,51]. Overall, the immune-inflammatory reaction positively correlated with the presence of preservative and its concentration, with the adjunctive evidence that the change of BAK with less toxic preservatives, such as polyquaternium-1, in part preserved follicles from the infiltration of inflammatory cells [52]. The effects of antiglaucoma drugs on human CALT in vivo are very limited, but the available reported information agrees with those described in animal models [25]. These reported findings may support the evidence that antiglaucoma medications induce an immune-related ocular surface disorder in the lymphoid structures related for the most part to the use of preservatives.

#### 3.2.2. Cornea

Topical IOP-lowering medications may induce significant modifications of corneal epithelial layers, sub-basal nerve plexus, stroma, and endothelium; all corneal changes can be detected with IVCM, with different confocal features positively correlated with clinical OSD symptoms [53].

Martone et al. [54] compared the long-term effects of PF and preserved antiglaucoma medications on the cornea in POAG and OH patients. They reported a reduced density of superficial epithelial cells in all groups of patients treated with preservative formulations, suggesting a toxic effect of BAK on these cells. The preservative groups also showed an increased density of basal epithelial cells, probably related to a proliferative stimulus induced from the superficial layer [54].

Medical therapy may also induce changes in corneal DCs, normally located within the limbal and central epithelia of the cornea [55]. In patients with glaucoma, the DCs’ density was reported to be two to three times higher at the limbus, and more than ten times higher in the central cornea, than that in healthy eyes. This density was also higher in multi-treated patients and in patients taking preserved drugs or with a high cumulative daily dose of BAK [55]. This was the first study that specifically investigated the DCs in the entire cornea in glaucomatous patients, suggesting that they may have a role in the development of the OSD. However, given the retrospective nature of the study, it was not clear whether the DCs’ density increase was primary related to the drugs’ toxicity or was a consequence of the iatrogenic dry eye [55].

As reported in these previous reports, the epithelium was the corneal layer more frequently altered by the antiglaucoma therapy. However, the inflammatory processes occurring within the epithelium have been also hypothesized to determine stromal changes through the promotion of apoptotic phenomena and increased stromal proteolytic activity [25,54].

Several studies have evaluated stromal reflectivity, keratocyte density, and sub-basal plexus nerves parameters such as density, tortuosity, and reflectivity; nerve fiber changes are crucial, as nerve fibers regulate the corneal trophism, which is essential to maintain a healthy ocular surface [56].

The increase in keratocyte density in the entire stroma was reported in patients under PGA therapy for at least three years, suggesting that PGA induces a disruption of the architecture of the extracellular matrix through the activation of metalloproteinases and inhibition of tissue inhibitors of metalloproteinases [57].

Different results were reported in a previous study of Baratz et al. [58]. They reported no significant differences in keratocyte density or corneal endothelial cells morphology between treated and untreated eyes. However, they showed a significant decrease in the density of sub-basal plexus nerves in the treated group of the Ocular Hypertension Treatment Study after six years of therapy. These contrasting results were probably related by the fact that the author [58] studied the actions of the glaucoma therapy in general without differentiating between the selective effects that each class of hypotensive drugs may have on the stroma.

The reduction in the number and density of sub-basal plexus nerves and an increased number of nerve beading and tortuosity at confocal microscopy were recognized in all therapy regimens [56], but were more evident in the case of treatment with preserved medications [54,59].

A recent study of Saini et al. reported a significant reduction in nerves’ number, length, and density in patients controlled with two or more topical antiglaucoma medications with preservatives (latanoprost 0.005%, brimonidine and timolol 0.5%) [60].

Inversely, PF formulations seem to be associated with less toxicity on the ocular surface. In 2013, Rossi et al. [61] conducted a prospective study to evaluate the corneal changes at IVCM after twelve months of therapy with PF tafluprost in naïve glaucoma patients and in patients switched to tafluprost from a previous different monotherapy. They showed that patients that switched from preservative monotherapy to tafluprost presented an improvement of several confocal parameters, such as epithelial cells’ density, keratocyte activation, number of sub-basal nerves, and nerve tortuosity, suggesting a possible reversible effect of the ocular surface toxicity [61].

In another prospective study, the same author performed IVCM of the cornea in three groups of patients: naïve patients treated with PF tafluprost 0.0015%; naïve patients treated with preserved bimatoprost 0.003% or preserved travoprost 0.004%; and patients on topical preserved prostaglandin or beta-blocker monotherapy that need a switch [62]. They found that naïve-to-therapy patients did not show statistically significant changes from baseline regarding nerves’ parameters such as number, tortuosity, and beading like formations, whereas the switch to PF tafluprost in patients on topical preserved monotherapy improved the nerve status [62].

However, different results, probably related to the different IVCM employed and treatment duration, were reported by Rolle et al. [63]. In a cross-sectional study, they found corneal alterations on patients treated with PF-tafluprost 0.0015% or PF-timolol 0.1% for three years, confirming that the active compound itself may have a toxic effect on the ocular surface.

Antiglaucoma drugs may also determine polymegathism, pleomorphism, and reduction in density of the corneal endothelial cells [56], and may affect the corneoscleral limbus in terms of worsening of transitional epithelium regularity, inflammation of Vogt’s palisades, and increase in DCs [55,64].

Mastropasqua et al. [64] studied the immunoinflammatory effects of antiglaucoma medications on the corneoscleral limbus and found that the irregularity of the transition epithelium, DCs’ activation, and fibrosis of the Vogt’s palisades around the entire limbal circumference were more common in patients receiving BAK-preserved drugs or controlled with multitherapy. These alterations were correlated with the increased expression of both human leucocyte antigen-DR and interleukin-6 at impression cytology, suggesting that the inflammation may be the main mechanism involved in the limbal alterations [64].

#### 3.2.3. Meibomian Glands

MGs are holocrine glands located in the tarsal plate of the eyelids and synthesize the meibum, a lipoid complex essential to stabilize the tear film and decrease its evaporation [65]. IVCM allows to identify MGs and to obtain several MG-related parameters of the orifice and acinus [65].

The main MGs’ modifications induced by antiglaucoma therapy are represented by the reduction of glandular density and area, signs of glandular loss and decreased meibum production, an increased reflectivity of the acinar secretion, dilation of the ductal orifice, and inhomogeneity of MGs’ interstice and wall, which indicate glandular inflammation [66].

These findings are more evident in the case of therapy with preserved drugs. Specifically, Agnifili et al. [67] reported that preserved PGAs were more toxic than the PF formulation, whereas no differences were found between preserved and unpreserved β-blockers; this suggested that, besides the detrimental effect of BAK, active compounds, and especially PGA, also play a role in inducing MGs’ alterations.

Additionally, MGs’ changes are even more frequent in patients treated with two or more drugs compared with patients in monotherapy. It has been shown that the prostaglandin/timolol fixed combinations induce less modifications MGs and GCs compared with the unfixed combination of latanoprost + timolol, and these changes are even more contained in the PF formulation of bimatoprost/timolol [68]. These results sustain the higher tolerability for the ocular surface of fixed combinations over the unfixed regimens. The modification of the therapy regimen should be strongly considered in the case of glaucoma-related OSD, as it may lead to a potential reversibility of damage of GCs and MGs. These aspects should be taken into account especially when patients require a multiple therapy to control the disease.

### 3.3. Morphologic Changes: Anterior Segment Optical Coherence Tomography

In the last years, AS-OCT has gained growing importance in studying all the anterior segment structures of the eye, providing information from a more macroscopic point of view compared with IVCM [69].

In the field of glaucoma, AS-OCT allows to visualize the aqueous outflow pathways, the anterior chamber angle anatomy, and function, and to evaluate the alterations induced by IOP-lowering agents on the ocular surface including corneal epithelia, tear film, and tear meniscus (Table 1 and Table 2) [37,70,71].

#### 3.3.1. Tear Meniscus

**Figure 2:**
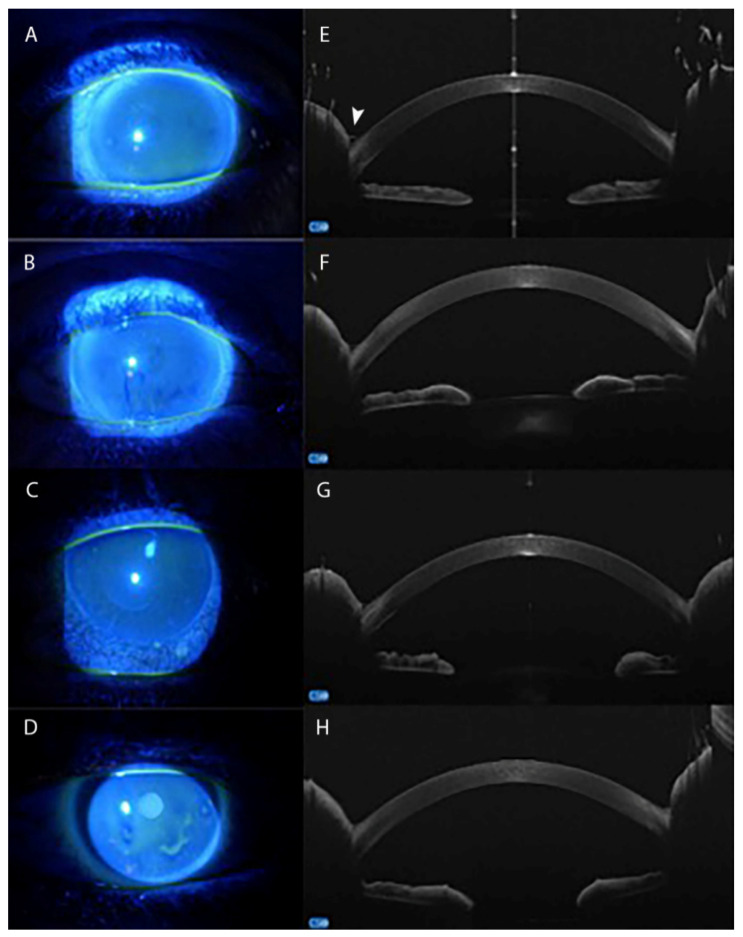
Anterior segment-optical coherence tomography (AS-OCT) of tear meniscus in medically controlled glaucoma. Fluorescein appearance of the tear meniscus and tear film in patients controlled with a preservative-free or preserved PGA mono-therapy (**A**,**B**), or with two or more drugs per day (**C**,**D**). AS-OCT shows the progressive reduction of the tear meniscus height (arrowhead) with increasing the number of medications, and the cumulative daily dose of preservative, required to control the disease (**E**–**H**).

More recently, studies have also used AS-OCT in clinical practice to evaluate the tear film and tear meniscus in patients affected by dry eye disease [72,73], with tear meniscus reduction being proposed as a biomarker of dry eye and, generally, OSD. However, to date, few data are available about the tear meniscus alterations, as detected at AS-OCT, in glaucomatous patients on medical therapy (Figure 2).

In a recent case-control observational study, Agnifili et al. [26] evaluated the tear meniscus morphometric modifications using AS-OCT in glaucomatous patients controlled with different therapy regimens. The authors observed that the tear meniscus height and area were significantly reduced in glaucoma patients compared with normal eyes. Moreover, no differences were detected between β-blockers and PGA monotherapy, where the presence of one or two preservative-containing eye drops did not modify the tear meniscus.

However, the tear meniscus height and area were significantly reduced in patients on multitherapy with two or more medications, with values similar to those observed in the case of dry eye [26]. These parameters were also positively correlated with ocular surface clinical tests such as break-up time, corneal fluorescein staining, and Schirmer Test I, and negatively with the ocular surface disease index questionnaire score, suggesting that the evaluation of tear meniscus at AS-OCT may allow to characterize some aspects of the glaucoma therapy-related OSD [26]. This was the first study conducted on patients with glaucoma; it was retrospective and included a small sample of patients. Therefore, it would be interesting to conduct further studies in order to confirm these results [26] and to differentiate the impact of the cumulative daily dose of preservative and the role of each active compound on the tear meniscus morphometry.

#### 3.3.2. Corneal Epithelial Thickness

The chronic use of antiglaucoma drugs induces corneal epithelial changes, which may also lead to macroscopic alterations, such as corneal epithelial thickness modification, which can be further considered as an additional feature of glaucoma therapy-related OSD [74]. The measure of corneal epithelial thickness using AS-OCT allows to obtain information about the ocular surface status in the early stages of the glaucoma therapy-related OSD [75].

In a retrospective study, Cennamo et al. [75] measured corneal epithelial thickness in glaucoma patients and evaluated changes in thickness based on the number of corneal microvilli using scanning electron microscopy. The authors showed that the early epithelial damage in patients on therapy with preservative glaucoma medications is characterized by corneal epithelial thickness reduction and expression of a cellular damage, which initially involves corneal epithelial microvilli. However, the prolonged use of drugs may induce an advanced damage of epithelial microvilli with epithelial sub-oedema and inflammatory cells’ infiltration, which finally determined an increase in corneal epithelial thickness [75]. However, this study did not take into consideration factors that could contribute to corneal epithelial damage such as treatment duration and types of antiglaucoma drugs and preservatives.

Batawi et al. [76] compared the epithelial, stromal, and central corneal thickness between POAG and healthy eyes assessed by AS-OCT that, differently from confocal microscopy and ultrasound pachimetry, can measure the corneal epithelium and stromal layer separately. The authors found that POAG patients had lower absolute stromal and total thickness values compared with healthy eyes. Moreover, thickness negatively correlated with the number of antiglaucoma medications, suggesting that the preservative amount may probably affect the corneal epithelium [76].

Differently, a recent study did not find significant differences in corneal epithelial thickness among patients under different types and durations of treatment, while the only factor influencing corneal epithelial thickness was represented by age [77]. In a similar study, Dogan et al. [78] reported that there was no significant correlation between corneal thickness and glaucoma type, duration of therapy, number of drugs, and daily drops.

**Table 1:** Summary of clinical studies evaluating the main morphological changes induced by beta-blocker and prostaglandin analogue monotherapy on the ocular surface using IVCM and AS-OCT.

Ocular Tissue	Authors	Year	Technique	Study Population	Specific Therapy (Preserved/Unpreserved)	Main Results
**Conjunctiva**	Ciancaglini M. et al. [38]	2008	IVCM ^a^	Untreated OH ^b^ POAG ^c^ Healthy controls	Preserved timolol 0.5%	In glaucoma patients with respect to healthy controls: − Evidence of conjunctival EM ^d^ in OH ^b^ and POAG ^c^ groups [38] − Increase in DCs’^e^ density and SFD ^f^ [47] Comparison between BB ^i^ and PGA ^j^ and between preserved and PF ^h^ medications: − Decrease of GCs’ ^l^ density [42,43,47] and high score of epithelial regularity in the preserved BB ^i^ group compared with the PF ^h^–BB ^i^ group − Increase of GCs’ ^l^ density in patients treated with PF ^h^ tafluprost [45], Bimatoprost 0.01% [46], and travoprost 0.004% [47]
					Preserved levobunolol 0.5%	
					Latanoprost 0.005%	
					Travoprost 0.004%	
					Bimatoprost 0.03%	
	Ciancaglini M. et al. [42]	2008	IVCM ^a^, IC ^g^	OH ^b^	Preserved levobunolol	
				POAG ^c^	PF ^h^ levobunolol	
	Mastropasqua L. et al. [45]	2013	LSCM ^k^, IC ^g^	POAG ^c^	Preserved latanoprost	
				Healthy controls	PF ^h^ tafluprost	
	Figus M. et al. [46]	2014	LSCM ^k^	POAG ^c^	Bimatoprost 0.01%	
					Bimatoprost 0.03%	
	Frezzotti P. et al. [43]	2014	IVCM ^a^	OH ^b^ POAG ^c^ Healthy controls	Preserved timolol	
					PF ^h^ timolol	
	Zhu W. et al. [47]	2015	IVCM ^a^	POAG ^c^	Carteolol hydrochloride 2%	
				Healthy controls	Travoprost 0.004%	
**Cornea**	Baratz K. et al. [58]	2006	IVCM ^a^	OH ^b^ (medication group) OH ^b^ (observation group)	timolol 0.5%	In glaucoma patients with respect to healthy controls: − Decrease in sub-basal plexus nerve density [56,58,63] − Decrease in endothelial cell density [56] − Increase in nerve tortuosity [56,63] − Increase in keratocyte stromal density [54,57] − Increase in stromal reflectivity [63] − Increase in DCs’ ^n^ density [55] − Decrease of corneal thickness measurements at AS-OCT ^r^ [75,76,77,78] Comparison between preserved and PF ^h^ medications: − Less confocal changes of the cornea parameters (epithelial, endothelial, and dendritic cells’ density; sub-basal nerve reflectivity; tortuosity; and beading) in glaucoma patients treated with PF ^h^ medications [55,59] and in patients on preserved monotherapy that switched to PF ^h^ therapy [54,55,61,62] − At limbus: higher limbal inflammation, irregularity of the transition epithelium, DCs’ ^n^ activation, and fibrosis of the Vogt’s palisades in patients receiving BAK ^q^ preserved drugs [64]
					betaxolol 0.25%	
					Latanoprost 0.005%	
					Unoprostone 0.15%	
	Martone G. et al. [54]	2009	IVCM ^a^	OH ^b^ POAG ^c^ Healthy controls	Preserved timolol 0.5%	
					PF ^h^ timolol 0.5%	
	Bergonzi C. et al. [57]	2010	IVCM ^a^	POAG ^c^	BB ^i^ (not specified)	
					PGA ^j^ (not specified)	
	Ranno S. et al. [56]	2011	IVCM ^a^	POAG ^c^	BB ^i^ (not specified)	
				Healthy controls	PGA ^j^ (not specified)	
	Rossi G.C.M. et al. [61]	2013	IVCM ^a^	OH ^b^ OAG ^m^ Healthy controls	PF ^h^ Tafluprost	
	Fogagnolo P. et al. [59]	2015	IVCM ^a^	OH ^b^ POAG ^c^ PXG ^o^ NTG ^p^	Unpreserved tafluprost 0.0015%	
					Preserved latanoprost 0.005% + BAK ^q^ 0.02%	
	Mastropasqua R. et al. [64]	2015	LSCM ^k^, IC ^g^	POAG ^c^ Sjogren syndrome-dry eye Healthy controls	Preserved timolol 0.5%	
					PF ^h^ timolol 0.5%	
					Preserved bimatoprost 0.001%	
					PF ^h^ tafluprost 0.015%	
	Mastropasqua R. et al. [55]	2016	IVCM ^a^	OAG ^m^ Sjogren syndrome-dry eye Healthy controls	Preserved timolol 0.5%	
					PF ^h^ timolol 0.5%	
					Preserved bimatoprost 0.001%	
					PF ^h^ tafluprost 0.015%	
	Rolle T. et al. [63]	2017	IVCM ^a^	OH ^b^ POAG ^c^ Healthy controls	PF ^h^ Timolol 0.1%	
					Tafluprost 0.0015%	
	Rossi G.C.M. et al. [62]	2019	IVCM ^a^	OH ^b^ OAG ^m^ Healthy controls	Preserved BB ^i^ (not specified)	
					PF ^h^ Tafluprost	
					Preserved bimatoprost 0.003%	
					Preserved travoprost 0.004%	
	Cennamo G. et al. [75]	2018	AS-OCT ^r^, SEM ^s^	OAG ^m^ Healthy controls	Preserved monotherapy (not specified)	
	Batawi H. et al. [76]	2018	AS-OCT ^r^	POAG ^c^	Timolol 0.005%	
				Healthy controls	Latanoprost	
	Montorio D. et al. [77]	2020	AS-OCT ^r^	POAG ^c^	BB ^i^ (not specified)	
				Healthy controls	PGA ^j^ (not specified)	
	Dogan E. et al. [78]	2020	AS-OCT ^r^	POAG ^c^ PACG ^t^ PXG ^o^ Healthy controls	BB ^i^ (not specified)	
					PGA ^j^ (not specified)	
**Tear Meniscus**	Agnifili L. et al. [26]	2020	AS-OCT ^r^	POAG ^c^ EDE ^u^ Healthy controls	BB ^i^ (not specified)	Decrease in TMH ^v^ and TMA ^w^ in glaucoma patients with respect to healthy subjects
					PGA ^j^ (not specified)	TMH ^v^ and TMA ^w^ negatively correlated with OSDI ^x^ score

^a^ IVCM = in vivo confocal microscopy; ^b^ OH = ocular hypertension; ^c^ POAG = primary open angle glaucoma; ^d^ EM = epithelial microcysts; ^e^ DCs = dendritic cells; ^f^ SFD = subepithelial collagen fiber diameter; ^g^ IC = impression citology; ^h^ PF = preservative free; ^i^ BB = beta-blocker; ^j^ PGA = prostaglandin analogue; ^k^ LSCM = laser scanning confocal microscopy; ^l^ GCs = goblet cells; ^m^ OAG = open angle glaucoma; ^n^ DCs = dendritic cells; ^o^ PXG = pseudoesfoliative glaucoma; ^p^ NTG = normal tension glaucoma; ^q^ BAK = benzalkonium chloride; ^r^ AS-OCT = anterior segment optical coherence tomography; ^s^ SEM = scanning electron microscopy; ^t^ PACG = primary angle closure glaucoma; ^u^ EDE = evaporative dry eye; ^v^ TMH = tear meniscus height; ^w^ TMA = tear meniscus area; ^x^ OSDI = ocular surface disease index.

**Table 2:** Summary of clinical studies evaluating the main morphological changes induced by combined therapy on the ocular surface using IVCM and AS-OCT.

Ocular Tissue	Authors	Year	Technique	Study Population	Specific Therapy	Main Results
**Conjunctiva**	Ciancaglini M. et al. [38]	2008	IVCM ^a^	Untreated OH ^b^ POAG ^c^ Healthy controls	Unfixed CT ^d^:	In the case of multitherapy with respect to monotherapy: − Higher EM ^e^ density and area [38] − Higher DCs’ ^f^ density [47] − Greater GCs’ ^g^ loss [41] Less MGs’ ^k^ and GCs’ ^g^ modifications in the case of fixed CT ^d^ and PF ^l^ formulation [68]
					latanoprost/timolol	
					travoprost/timolol	
					bimatoprost/timolol	
	Zhu W. et al. [47]	2015	IVCM ^a^	POAG ^c^	CT ^d^:	
				Healthy controls	Two or three drugs (not specified)	
	Di Staso S. et al. [41]	2018	IVCM ^a^	POAG ^c^ PXG ^h^ PG ^i^ DED ^j^ Healthy controls	Fixed CT ^d^:	
					latanoprost/timolol	
					travoprost/timolol	
					bimatoprost/timolol	
					Unfixed CT ^d^:	
					bimatoprost/timolol	
	Agnifili L. et al. [68]	2018	IVCM ^a^	POAG ^c^	Fixed CT ^d^:	
				PXG ^h^	prostaglandin/timolol	
				PG ^i^	Unfixed CT ^d^:	
				Healthy controls	latanoprost + timolol	
**Cornea**	Baratz K. et al. [58]	2006	IVCM ^a^	OH ^b^ (medication group)	Fixed CT ^d^:	In the case of multitherapy with respect to healthy controls: − Decrease in central corneal sub-basal nerve fiber number, length, and density and increase in basal epithelial cells’ density [60] − Decrease of corneal thickness measurements at AS-OCT ^o^ [77,78] In the case of multitherapy with respect to monotherapy: − Higher decrease in sub-basal plexus nerve density [58] − Higher DCs’ ^f^ density [55] − At limbus: higher limbal inflammation, irregularity of the transition epithelium, DCs’ ^f^ activation, and fibrosis of the Vogt’s palisades [64] − Discording results in the corneal thickness parameters at AS-OCT ^o^ with regard to the number of drugs used [76,77,78]
				OH ^b^ (observation group)	Dorzolamide/timolol	
	Mastropasqua R. et al. [64]	2015	LSCM ^m^ IC ^n^	POAG ^c^ Sjogren syndrome-dry eye Healthy controls	Fixed CT ^d^:	
					preserved latanoprost/timolol	
					preserved dorzolamide/timolol	
					preserved bimatroprost/brimonidine/timolol	
					preserved latanoprost/dorzolamide/timolol	
					Unfixed CT ^d^:	
					preserved bimatoprost/timolol	
					preserved brimonidine/timolol	
	Mastropasqua R. et al. [55]	2016	IVCM ^a^	OAG ^p^ Sjogren syndrome-dry eye Healthy controls	Fixed CT ^d^:	
					preserved latanoprost/timolol	
					preserved dorzolamide/timolol	
					preserved brimonidine/timolol	
					preserved bimatroprost/brimonidine/timolol	
					Unfixed CT ^d^:	
					preserved bimatoprost/timolol	
	Saini M. et al. [60]	2017	IVCM ^a^	OH ^b^ POAG ^c^ PACG ^q^ JOAG ^r^ NTG ^s^ Healthy controls	Preserved CT ^d^:	
					timolol/brimonidine	
					timolol/latanoprost	
					latanoprost/brimonidine	
	Batawi H. et al. [76]	2018	AS-OCT ^o^	POAG ^c^ Healthy controls	CT ^d^ (not specified)	
	Montorio D. et al. [77]	2020	AS-OCT ^o^	POAG ^c^ Healthy controls	CT ^d^ (not specified)	
	Dogan E. et al. [78]	2020	AS-OCT ^o^	POAG ^c^ PACG ^q^ PXG ^h^ Healthy controls	CT ^d^ (not specified)	
**Tear Meniscus**	Agnifili L. et al. [26]	2020	AS-OCT ^o^	POAG ^c^ EDE ^t^ Healthy controls	Two or more drugs (not specified)	Smaller TMH ^u^ and TMA ^v^ in the case of multitherapy with respect to monotherapy

^a^ IVCM = in vivo confocal microscopy; ^b^ OH = ocular hypertension; ^c^ POAG = primary open angle glaucoma; ^d^ CT = combined therapy; ^e^ EM = epithelial microcysts; ^f^ DCs = dendritic cells; ^g^ GCs = coblet cells; ^h^ PXG = pseudoesfoliative glaucoma; ^i^ PG = pigmentary glaucoma; ^j^ DED = dry eye disease; ^k^ MGs = Meibomian glands; ^l^ PF = preservative free; ^m^ LSCM = laser scanning confocal microscopy; ^n^ IC = impression citology; ^o^ AS-OCT = anterior segment optical coherence tomography; ^p^ OAG = open angle glaucoma; ^q^ PACG = primary angle closure glaucoma; ^r^ JOAG = juvenile open angle glaucoma; ^s^ NTG = normal tension glaucoma; ^t^ EDE = evaporative dry eye; ^u^ TMH = tear meniscus height; ^v^ TMA = tear meniscus area.

## 4. Surgical Treatment

### 4.1. Ocular Surface and Surgical Therapy

Surgical treatment is considered when medical therapy is not able to control the IOP and to stabilize the progression of the disease or in the case of hypersensitive reactions to antiglaucoma drugs [79].

Trabeculectomy is the gold standard and the most effective filtering procedure. It is characterised by the formation of an intrascleral fistula draining the aqueous humor from the anterior chamber to the subconjunctival space, which is commonly referred to as a filtering bleb [80]. However, trabeculectomy may widely alter the ocular surface, causing a persistent clinical or subclinical inflammatory process [30,81].

Studies have demonstrated that patients with glaucoma filtering bleb may experience ocular discomfort in terms of burning, tearing, dry eye like signs, pain, and dysesthesia [14,82,83]. These symptoms may be related to the formation and location of the bleb, the application of anti-mitotic agents, and the post-operative wound-healing processes within the conjunctiva that may contribute to the ocular surface changes [40,84].

On the other hand, the cessation of chronic use of IOP-lowering agents and the topical instillation of steroids after surgery may have a positive effect on the ocular surface status, which is probably related to the end of the chronic inflammation and the toxic action of the medications [84].

Recently, new surgical procedures called minimally-invasive glaucoma surgeries (MIGS) have been introduced in order to reduce the invasiveness of conventional filtering surgery. They may be considered an appropriate choice for patients with concomitant OSD and glaucoma [16]. These procedures spare or induce minimal trauma on the conjunctiva, resulting in a reduction of the post-operative inflammatory reaction and in less irritation to the ocular surface [85].

### 4.2. Morphologic Changes: In Vivo Confocal Microscopy

**Figure 3:**
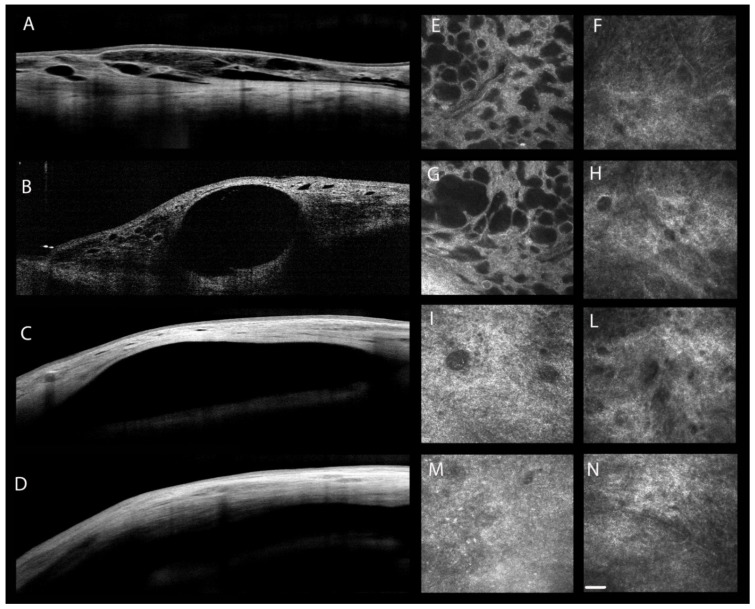
AS-OCT and IVCM conjunctival bleb features after glaucoma filtration surgery. (**A**–**D**) Filtration bleb imaged by AS-OCT. Diffuse (**A**) or cystic (**B**) filtration bleb after completely successful trabeculectomy, showing numerous, differently sized hypo-reflective spaces filled with aqueous humor; incapsulated (**C**) and flat (**D**) filtration bleb after a failed trabeculectomy without evidence of hypo-reflective intra-bleb wall spaces. (**E**–**N**) Bleb-wall imaged by IVCM. In diffuse (**E**,**F**) or cystic (**G**,**H**) functioning filtration blebs, the bleb-wall epithelium (**E**,**G**) shows several microcysts with a loosely arranged stroma (**F**,**H**), indicating a good aqueous humor percolation and a minimal hydraulic resistivity through the bleb-wall layers. Opposite features are present in incapsulated (**I**,**L**) or flat (**M**,**N**) non-functioning filtration blebs: the bleb-wall epithelium (**I**,**M**) shows rare microcysts, whereas the stroma (**L**,**N**) appears densely arranged. These features indicate an inefficient aqueous humor percolation through the bleb-wall layers. Bar represents 50 µm.

In the glaucoma surgical field, IVCM is applied to study the trans-scleral aqueous outflow, to differentiate functioning from non-functioning conjunctival filtering blebs, and to follow the conjunctival wound-healing process [86] (Table 3 and Figure 3). The IVCM use after filtering surgery would be appropriate in order to help clinicians in identifying the early signs of failure and to adopt appropriate procedures, such as laser suture lysis or needling with or without anti-metabolite injection, before the IOP rises and the bleb fails.

Non-functioning bleb presents a hyper-reflective subepithelial tissue with dense collagenous connective tissue and blood vessels and few or no epithelial microcysts [25,87,88,89].

Inversely, a functioning filtering bleb is characterized by a normal conjunctival epithelium with a hypo-reflective subepithelial tissue and numerous epithelial microcysts, which are considered as in vivo indicators of the trans-bleb aqueous humor passage [87,90,91].

At IVCM, epithelial microcysts appear as round or oval-shaped optically clear structures that may contain inflammatory cells and amorphous material and are surrounded by a hyper-reflective wall [40].

Ciancaglini et al. [90] evaluated the IVCM characteristics of the bulbar conjunctiva in POAG patients before and one month after trabeculectomy. They reported a fivefold increase in conjunctival epithelial microcysts’ density and area in correspondence to the bleb after surgery suggesting that is probably related to the enhancement of the aqueous filtration through the sclera and conjunctiva [90].

On the conjunctival surface of functioning bleb, the presence of a great number of atypical GCs may be also observed, which appear as hypo-reflective structures that show weak or no mucin marker 5AC immunostaining at impression cytology [92]. The epithelial microcysts seem to correspond to these GCs containing aqueous humor, suggesting that the transconjunctival aqueous outflow through the bleb wall epithelium occurs at the level of GCs [93].

In a prospective, cross-sectional study, Agnifili et al. [93] correlated the preoperative conjunctival GCs’ density and mucin positivity, using IVCM and impression cytology, with the 12-month success in patients undergoing trabeculectomy. The authors reported higher postoperative values of microcysts’ density and area in patients with a successful surgery compared with failed cases. A strong, positive correlation was found between these parameters and the baseline GCs’ density and the mucin marker 5AC, thus confirming a direct active role of GCs in vehiculating the aqueous humor [93].

Based on these reported findings, it is interesting to underline that the surgical success could also depend on the GCs’ density available before surgery. Therefore, it is mandatory to suggest a careful selection of the glaucoma medications used in the medical management of glaucoma in order to preserve the integrity of this critical cell population.

Other preoperative conjunctival parameters (as expression of the inflammatory status of the conjunctiva) were considered as predictive tools for the surgery outcome. Mastropasqua et al. [94] reported that high preoperative levels of DCs, low levels of GCs, and a hyper-reflective stroma in correspondence to the site of surgery significantly increased the risk of bleb dysfunction and failure.

In a recent six-month, prospective, case-control study, Agnifili et al. [84] compared the in vivo and ex vivo characteristics of the ocular surface before and after successful filtration surgery. They showed an increase in GCs’ density and a reduction in MGs’ density, MGs’ inhomogeneity, limbal DCs’ density, sub-basal corneal nerve inhomogeneity, and conjunctival human leucocyte antigen-DR positivity in the surgical group compared with the medical control group [84]. Based on these findings, the authors concluded that the ocular surface improved after glaucoma filtration surgery [84].

The filtering procedure may also induce different stromal features according to the bleb filtering ability. Functioning filtering bleb shows a high density of stromal cystic spaces, absence of encapsulated cysts in the stroma, small diameters, and less tortuosity of stromal vessels [95,96]. As for epithelial microcysts, stromal cystic spaces were considered as a sign of aqueous humor passage through the bleb wall [25]. Conversely, early unfavorable signs are few cystic stromal spaces and large vessel diameters [95]. Moreover, Guthoff et al. [95] showed that functioning bleb presented a trabecular or reticular stromal pattern, whereas non-functioning bleb is characterized by a compacted or corrugated stromal pattern.

Additionally, conjunctival and stromal modifications at the bleb site may be determined by the use of anti-fibrotic agents during surgery.

Ciancaglini et al. [88] did not report any microscopic differences between patients who received intraoperative mitomycin C (MMC) and patients who did not. However, different studies showed the presence of hyper-reflective microdots in the superficial epithelium layer, which probably represented necrotic or inflammatory cells, and the presence of a rarified stroma with large cystic non-encapsulated spaces in the case of MMC administration [87,96].

Finally, only a few studies were performed regarding the effects of MIGS on the ocular surface [85,97,98].

A one-year prospective study of Fea et al. [97] evaluated the efficacy, safety, and bleb morphology following implantation of XEN 45 gel stent combined or not with cataract surgery. The authors reported an increase in mean microcysts’ area and density associated with a reduction of density of subepithelial connective tissue in the postoperative period [97]. Additionally, the stromal density was significantly lower in the case of successful surgery. These findings could suggest that the stent implantation may induce a new or increase an alternative aqueous humor outflow [97].

Moreover, Sacchi et al. [98] retrospectively analyzed the conjunctival filtering bleb characteristics at IVCM after XEN gel implantation and trabeculectomy. IVCM showed a greater mean microcysts area in the case of trabeculectomy, suggesting that the larger scleral ostium obtained with this procedure led to a greater aqueous outflow towards the subconjunctival space [98].

Recently, Baiocchi et al. [85] compared the IVCM characteristics in glaucoma patients treated with XEN 45 gel stent implant, trabeculectomy, or medical therapy. In the case of XEN 45 Gel Stent in the subconjunctival space, the IVCM documented the presence of regular corneal epithelial cells with microcysts and normo-reflective subepithelial nerve plexus. These findings are an expression of a lower rate of ocular surface inflammation. Differently, the combined procedure determined a higher inflammation rate that was also more pronounced in the case of trabeculectomy or medical therapy [85]. Therefore, from the results of this study, we may suggest that the XEN implant may be a good and alternative option in the hypertensive glaucoma management. This surgery, if well timed, could represent a solution in patients where a successful surgical outcome after trabeculectomy could be compromised by severe ocular surface inflammation due to long-term antiglaucoma topical therapy.

### 4.3. Morphologic Changes: Anterior Segment Optical Coherence Tomography

AS-OCT provides a high resolution, objective, macroscopic detailed evaluation of the filtering bleb morphology and function after glaucoma surgery by analyzing, in a non-invasive manner, the conjunctival and subconjunctival characteristics (Table 3) [30]. It may be also used to determine which blebs are suitable for needling [99], to analyze bleb changes after laser suture lysis [100,101], and to predict the functioning of bleb at a later stage or the need to plan a bleb revision surgery [102,103]. Despite this consolidated knowledge about the AS-OCT conjunctival and subconjunctival changes following glaucoma surgery, at this moment, there is a lack of information regarding the ability of AS-OCT to detect the effects of surgery on the tear meniscus and corneal epithelia.

Generally, AS-OCT macroscopic bleb features after surgery are in agreement with microscopic characteristics as seen with confocal microscopy; however, these techniques have a different target in the filtering bleb analysis [25,88].

Different post-trabeculectomy bleb parameters are analyzed using AS-OCT including length and height of the inner bleb space, maximum and minimum bleb wall thickness, bleb wall density, subepithelial connective tissue density, and number of microcysts [88,104].

Features of bleb morphology associated with successful IOP-lowering are reported in many studies (Figure 3) [88,102,104,105,106]. Functioning filtering blebs are characterized by an increased bleb height, high density of epithelial microcysts, few conjunctival fibrosis, large internal fluid-filled cavity, thick bleb wall, and low reflectivity appearance of the bleb wall that may be an expression of loose connective tissue [88,103,106,107]. Conversely, failed blebs show opposite features. Moreover, the reflective appearance of the bleb wall and the width of filtration opening analyzed by AS-OCT are considered important predictors of post-trabeculectomy outcomes [108].

AS-OCT has been also performed to study the bleb morphology after MIGS [97,98,109,110,111].

Two prospective 12-month studies analyzed the bleb morphology and reflectivity after XEN gel stent implant and reported that patients with a low bleb-wall reflectivity were more likely to have a good surgical outcome [97,109]. Furthermore, blebs with subconjunctival separation morphology; diffuse distribution of fluid; and small, diffuse, and hypo-reflective cysts were associated to a low risk of failure [110].

Finally, two recent studies compared the macroscopic conjunctival filtering bleb features after XEN gel stent implant and trabeculectomy [98,111]. The filtering blebs formed when using the XEN gel stent are smaller and with fewer intra-bleb cystic cavities, and are associated with less fibrosis and a thicker epithelium than the trabeculectomy blebs [111]. Moreover, the XEN bleb showed a small area of bleb-wall sub-epithelium cyst-like structure; expression of the smaller scleral ostium obtained with the 45 µm lumen of the XEN gel implant compared with the trabeculectomy; and a low bleb wall reflectivity, which is a surrogate sign of fibrosis strongly correlated with the surgical outcome [98].

These results should be taken into consideration, indicating that each type of surgery is associated with specific morphological features of the filtration bleb. Therefore, the knowledge of these aspects detected at AS-OCT may help a clinician to predict the risk of filtration failure in the very early post-operative period.

### 4.4. Vascular Changes: Optical Coherence Tomography Angiography

Recently, optical coherence tomography angiography (OCT-A) has been gaining a role in post-operative filtration bleb assessment. OCT-A provides, in a quick and non-invasive way, information about the bleb-wall vasculature that cannot be adequately explored with the available ophthalmologic imaging platforms [112,113].

The bleb vascularity is related to the conjunctival wound-healing process; a higher conjunctival vascularization after surgery is, in fact, correlated with higher levels of inflammation and increased risk of filtration failure [112,114].

Few studies have evaluated the relationship between filtering bleb vascularization at OCT-A and surgical success after glaucoma surgery (Table 3) [112,113,114,115,116].

Yin et al. [112] explored the association between the vessel area in the operative region and the surgical outcomes post-trabeculectomy. They reported a positive correlation between the vessel area at one month and the IOP at six months after trabeculectomy [112].

Similar results were presented by Hayek et al. [115]. They found a significant correlation between preoperative conjunctival vascular density and mean IOP at six months postoperatively. Specifically, a low preoperative conjunctival vessel density on the surgical site was associated with a lower post-surgical IOP, fewer needlings, and less use of IOP-lowering drugs [115].

Moreover, other OCT-A features such as the colour and brightness densities of the bleb-wall exhibited a good correlation with the vessel grading using conventional clinical bleb grading systems, suggesting that these OCT-A parameters may be considered as biomarkers of the bleb vascularity and outcome after surgery [116]. Seo et al. [114] compared bleb vascularity parameters at OCT-A between MMC-augmented trabeculectomy and phacotrabeculectomy. They found differences in the conjunctival vascularity density according to the type of surgery. In the phacotrabeculectomy group, the bleb color and brightness densities were significantly higher than in the trabeculectomy group, suggesting that the combined procedure may probably lead to increased inflammation, which may lead to changes in vessels on the bleb [114].

Finally, Mastropasqua et al. [113] evaluated the filtration bleb features after XEN gel stent implantation using OCT-A. Successful filtration blebs presented a lower bleb wall vessel density and numerous and large areas of vessel displacement within the bleb wall. The authors suggested that these OCT-A signs may be considered angiographic biomarkers of a good aqueous humour percolation through the bleb wall layers, which can help to distinguish functioning from non-functioning filtering blebs [113].

**Table 3:** Summary of clinical studies evaluating the main features of functioning glaucoma filtering bleb using IVCM, AS-OCT, and OCT-A.

Authors	Year	Technique	Study Population	Surgical Procedure	Main Results
Labbè A. et al. [87]	2005	IVCM ^a^	OAG ^b^	Trabeculectomy	Functioning filtering bleb at IVCM ^a^: − increase in conjunctival EM ^d^ density and area [87,88,89,90,93,97] − low density of connective tissue [88,91,97] − trabecular or reticular stromal pattern with high density of stromal cystic spaces [95] − small diameters and less tortuosity of stromal vessels [95,96] − evidence of numerous atypical GCs ^i^ with weak or no mucin marker 5AC immunostaining [92,93] − less signs of inflammation in the case of XEN 45 gel stent implant with respect to combined procedure, trabeculectomy, or medical therapy [85] Functioning filtering bleb at AS-OCT ^j^: − low reflectivity appearance of the bleb wall [88,97,109,110] − cystic pattern [109,110] − few conjunctival fibrosis [88] − diffuse distribution of fluid and small, diffuse, and hyporeflective cysts [110] − Thinner and flatter hypo-reflective bleb wall and fewer intra-bleb cystic cavities in the case of XEN 45 gel stent implant with respect to trabeculectomy bleb [98,111] At OCT-A ^l^: − rarefied vascular network with low vessel density and numerous and large vessel displacement areas within the bleb-wall [113] − positive correlation between conjunctival vessel density and area and IOP ^m^ after surgery [112,115] − positive correlation between the colour and brightness densities of the bleb wall after surgery and the conventional vascular score bleb grading systems [116]. Colour and brightness densities of the bleb wall were significantly higher in the case of the combined procedure [114] Opposite features in the case of non-functioning filtering bleb.
Guthoff R. et al. [95]	2006	IVCM ^a^	OAG ^b^ CACG ^c^	Trabeculectomy	
Messmer E. et al. [96]	2006	IVCM ^a^	POAG ^e^ PXG ^f^ NTG ^g^ Secondary glaucoma	Trabeculectomy	
Amar N. et al. [92]	2008	IVCM ^a^ IC ^h^	OAG ^b^	Trabeculectomy	
Ciancaglini M. et al. [88]	2008	IVCM ^a^ AS-OCT ^j^	POAG ^e^ PXG ^f^	Trabeculectomy	
Sbeity Z. et al. [89]	2009	IVCM ^a^	POAG ^e^ Secondary glaucoma	Trabeculectomy	
Ciancaglini M. et al. [90]	2009	IVCM ^a^	POAG ^e^	Trabeculectomy	
Morita K. et al. [91]	2012	IVCM ^a^ UBM ^k^	POAG ^e^ PXG ^f^	Trabeculectomy	
Agnifili L. et al. [93]	2016	IVCM ^a^ IC ^h^	OAG ^b^ Healthy controls	Trabeculectomy	
Fea A. et al. [97]	2017	IVCM ^a^ AS-OCT ^j^	POAG ^e^	XEN 45 gel stent implant	
Olate-Perez A. et al. [109]	2017	AS-OCT ^j^	POAG ^e^	XEN 45 gel stent implant + cataract surgery	
Yin X. et al. [112]	2018	OCT-A ^l^	Primary Glaucoma	Trabeculectomy	
Hayek S. et al. [115]	2019	OCT-A ^l^	OAG ^b^ CACG ^c^	Trabeculectomy	
Lenzhofer M. et al. [110]	2019	AS-OCT ^j^	OAG ^b^	XEN 45 gel stent implant	
Teus M.A. et al. [111]	2019	AS-OCT ^j^	POAG ^e^ Healthy controls	XEN 45 gel stent implant Trabeculectomy	
Seo J.H. et al. [116]	2019	OCT-A ^l^	POAG ^e^ Secondary glaucoma	Trabeculectomy	
Seo J.H. et al. [114]	2019	OCT-A ^l^	POAG ^e^ PACG ^n^ Secondary glaucoma	Trabeculectomy Phacotrabeculectomy	
Baiocchi S. et al. [85]	2020	IVCM ^a^	POAG ^e^	XEN 45 gel stent implant Trabeculectomy	
Sacchi M. et al. [98]	2020	AS-OCT ^j^ IVCM ^a^	POAG ^e^ PXG ^f^ NTG ^g^	XEN 45 gel stent implant Trabeculectomy	
Mastropasqua R. et al. [113]	2020	OCT-A ^l^	POAG ^e^ PXG ^f^ PG ^o^	XEN gel stent implant	

^a^ IVCM = in vivo confocal microscopy; ^b^ OAG = open angle glaucoma; ^c^ CACG = chronic angle closure glaucoma; ^d^ EM = epithelial microcysts; ^e^ POAG = primary open angle glaucoma; ^f^ PXG = pseudoesfoliative glaucoma; ^g^ NTG = normal tension glaucoma; ^h^ IC = impression citology; ^i^ GCs = goblet cells; ^j^ AS- OCT = anterior segment optical coherence tomography; ^k^ UBM = ultrasound biomicroscopy; ^l^ OCT-A = optical coherence tomography angiography; ^m^ IOP = intraocular pressure; ^n^ PACG = primary angle closure glaucoma; ^o^ PG = pigmentary glaucoma.

## 5. Conclusions

Several studies have demonstrated the direct effects of glaucoma medical and surgical therapy on the tissues composing the ocular surface system.

New diagnostic tools such as IVCM and AS-OCT have progressively gained importance in the diagnosis and monitoring of glaucoma-related OSD. They allow to define, in a non-invasive way, the micro- and macrostructural changes of the ocular surface induced by medical therapy and surgical procedures, in order to detect the early signs of ocular surface modifications and the relationship between the ocular surface health status and the failure of filtering surgery.

The introduction of new drug formulations, slow-release devices, and MIGS procedures may induce less ocular surface toxicity, resulting in a positive effect on the patient’s QoL and satisfaction. Therefore, the coexistence of glaucoma and OSD should have been taken into account by physicians in order to choose the right therapy regimen and the suitable surgical timing and procedure.

## Data Availability

No new data were created or analyzed in this study. Data sharing is not applicable to this article.

## Abbreviations

AS-OCT	anterior segment optical coherence tomography
BAK	benzalkonium chloride
CALT	conjunctiva-associated lymphoid tissue
DCs	dendritic cells
GCs	goblet cells
IOP	intraocular pressure
IVCM	in vivo confocal microscopy
MGs	Meibomian glands
MIGS	minimally-invasive glaucoma surgeries
MMC	mitomycin C
OCT	optical coherence tomography angiography
OH	ocular hypertension
OSD	ocular surface disease
PF	preservative-free
PGA	prostaglandin analogs
POAG	primary open angle glaucoma
QoL	quality of life
